# Identification of potential inhibitors of SARS-CoV-2 S protein–ACE2 interaction by *in silico *drug repurposing

**DOI:** 10.12688/f1000research.52168.1

**Published:** 2021-05-07

**Authors:** Fabiola E Tristán-Flores, Diana Casique-Aguirre, Raquel Pliego-Arreaga, Juan A Cervantes-Montelongo, Ponciano García-Gutierrez, Gerardo Acosta-García, Guillermo A Silva-Martínez

**Affiliations:** 1 Ciencias Básicas, Tecnológico Nacional de México en Celaya, Celaya, Guanajuato, 38010, Mexico; 2 Escuela Nacional de Ciencias Biológicas (ENCB), Instituto Politécnico Nacional (IPN), CDMX, CDMX, 11340, Mexico; 3 Escuela de Medicina, Universidad de Celaya, Celaya, Guanajuato, 38080, Mexico; 4 Ingeniería Bioquímica, Tecnológico Nacional de México en Celaya, Celaya, Guanajuato, 38010, Mexico; 5 Departamento de Química, Universidad Autónoma Metropolitana–Iztapalapa, CDMX, CDMX, 09340, Mexico; 6 Ingeniería Bioquímica, Cátedras CONACYT-Tecnológico Nacional de México en Celaya, Celaya, Guanajuato, 38010, Mexico

**Keywords:** COVID-19, SARS-CoV-2, ACE2, Molecular Docking, Drug Repurposing

## Abstract

**Background: **Severe acute respiratory syndrome coronavirus 2 (SARS-CoV-2), a new coronavirus discovered that appeared in Wuhan, China, in December 2019, causes COVID-19 disease which have resulted in cases similar to SARS-atypical pneumonia. As of March 1, 2021, Mexico had reached 2.11 million cases of COVID-19 and 189 thousand deaths; around 116 million cases and 2.57 million deaths are reported worldwide with new cases and increasing mortality every day. To date, there is no specific commercial treatment to control the infection. Repurpose drugs targeting the angiotensin-converting enzyme 2 (ACE2) receptor represents an alternative strategy to block the binding of SARS-CoV-2 protein S and forestall virus adhesion, internalization and replication in the host cell.

**Methods:** Rigid molecular docking was performed using receptor binding domain of the S1 subunit of S protein (RBD _S1_)-ACE2 (PDB ID: 6VW1) interaction site and 1,283 drugs FDA approved and prescribed by the Mexican Public Health System. The results were analyzed by docking score, frequency of the drug in receptor site and the types of interactions at the binding site residues.

**Results:** About 40 drugs were identified as a potential inhibitor of RBD _S1_-ACE2 interaction. Within the top-ranked drugs, we identified ipratropium, formoterol and fexofenadine, which stands out as they are used as therapies to treat chronic obstructive pulmonary disease, asthma and virtually any respiratory infection.

**Conclusions**: Our results will serve as the basis for *in vitro* and *in vivo* studies to evaluate the potential use of those drugs to generate affordable and convenient therapies to treat COVID-19.

## Introduction

Emerging viruses can be defined as those whose incidence has increased in the last twenty years or whose presence has a high probability of increasing in the near future. Diseases caused by emerging viruses are one of the biggest public health threats globally. Some of the viruses that fall within this catalog are the avian influenza virus subtype H5N1, severe acute respiratory syndrome (SARS), Ebola, Zika, and MERS-CoV, to name a few ^1^. Coronaviruses (CoVs) are classified into four genera, α-CoV, β-CoV, γ-CoV, and δ-CoV2. α and β infect mammals, γ birds and δ birds and mammals, respectively ^2^. These viruses are of public health importance because they cause enteric, renal, and neurological respiratory diseases that range from asymptomatic to fatal ^3, 4^.

Severe acute respiratory syndrome coronavirus 2 (SARS-CoV-2) appeared in Wuhan, China, in December 2019, causing cases of SARS-like atypical pneumonia ^5, 6^, with a clinical picture of fever, general malaise, dry cough, shortness of breath and was called the coronavirus disease 2019 (COVID-19) ^7^. It can be asymptomatic, develop mild-to-severe symptoms, or cause death in elderly individuals or in patients with chronic diseases, such as hypertension, diabetes, and obesity ^8^. On January 31 ^st^ 2020, the World Health Organization (WHO) declared COVID-19 a public health emergency of international concern and, on March 12 ^th^, it was declared a global pandemic ^9^. In Mexico, local transmission (phase 2 of transmission) was declared on March 24 ^th^ 2020, which resulted in the suspension of non-essential activities in the country, generating economic losses in addition to public health problems and deaths associated with the disease. As of March 1 ^st^ 2021, Mexico had reached 2.1 million cases of COVID-19 and 189 thousand deaths; around 63.6 million cases and 1.47 million deaths have been reported worldwide.

To date, there is no specific commercial treatment to control the infection ^10– 12^. Measures such as early detection, blocking the route of transmission through social isolation, isolation of suspected cases, disinfection of objects, as well as frequent hand washing with soap, in addition to the use of biosafety equipment such as surgical masks for health personnel, which has been effective in reducing the transmission of COVID-19.

Coronaviruses, such as SARS-CoV-2, are positive-stranded RNA viruses enveloped on a membrane. The coronaviral genome is composed of approximately 30,000 nucleotides containing the envelope (E), membrane (M), spike (S), nucleocapsid (N) and ORFs *,* that encode non-structural proteins, including enzymes that appear during their in-host reproductive cycle-genes ^13^.

This virus measures 70 to 100 nm and belongs to the genus β-CoV ^14^ and it has been proposed that any of the aforementioned proteins that make up CoVs may be targets for the development of vaccines or drugs ^4^. Protein S plays an essential role on COVID-19 infection as it mediates the internalization on host cell and for the spread of the virus in the infected host ^15, 16^. This starts when the receptor binding domain of the S1 subunit (RBD _S1_) of S protein binds to the peptidase domain of angiotensin-converting enzyme 2 receptor (ACE2) ^17^ and it is know that disrupting the binding of S protein to ACE2 prevents the attaching an the later internalization of the virus to the host cell ^18^.

This protein interaction has recently been crystallized and deposited in the Protein Data Bank database ^19^, allowing us to use it as a model of study to test different strategies to counter SARS-CoV-2 infection, like blocking S glycoprotein-ACE2 interaction through the discovery of sites of potential pharmaceutical interest.

In 2019, Research and Development (R&D) spending in the pharmaceutical industry totaled 186 billion U.S. dollars globally and its projected to reach 233 billion U.S. dollars to 2026. Unfortunately, drug development takes large time and financial resources that not all countries possess, especially developing countries, like Mexico.

In this sense, drug repurposing or repositioning allow us to integrate all evidence, pharmacodynamics/kinetics, bioavailability, among other important parameters, from an existing and approved drug in order to manage emerging diseases, like COVID-19. All this translates into a considerable decrease in research time and investment of resources in R&D.

Different approaches have been taken in order to disrupt SARS-CoV-2 protein S-ACE2 interaction, as an example, many works has focus on finding potential biding sites on protein S structure, however, new variant strains has been detected worldwide, like B117 in UK, P1351 in South Africa, P1 and P2 in Brazil. All variant strains display the N501Y mutation, which is located on the RBD of the S protein, making the interaction more effective. In this sense, targeting RBD may be a transitory approach, therefore, an alternative strategy would be aiming at the ACE2 receptor. Some authors have pointed out some concerns about using drugs that targeting the renin–angiotensin signaling (RAS) pathway ^20– 22^, but Jia and collaborators ^23^ highlight current efforts of exploiting ACE2 as therapeutic target, like the use of pseudo-ligands to dominate the binding site for SARS-CoV-2 as an example. Therefore, inhibition of the SARS-CoV-2 protein S-ACE2 interaction trough aiming ACE2 receptor it is a plausible strategy. In this study, we screened a library consisting of 1300 FDA-approved drugs and acquired by Ministry of Health of Mexico in order to identify potential inhibitors of SARS-CoV-2–ACE2 interaction.

## Methods

### Molecule selection for docking

In order to run the docking simulation, we selected the X-ray crystal structure of SARS-CoV-2 RBD _S1_ in a complex with the ACE2 (PDB ID: 6VW1) which makes it have a more compact conformation in comparison with the SARS-CoV-2 RBD alone ^19^. Chains B, E, F, and oligosaccharides and crystallographic water molecules were removed from the system; hydrogen atoms were added, and the receptor structure was protonated at pH 7.0 and 300 K using the Protonate 3D tool. Partial charges were assigned using the Amber10:EHT forcefield. Later, the protein structure was subjected to energy minimization using the same forcefield. Site Finder MOE was employed to analyze potential binding sites near to the receptor binding ridge on ACE2 and docking simulation were carried out. The docking parameters were set to take each ligand conformation as unique molecule, using the Alpha Triangle algorithm as placement method (at least 100 different orientations or poses on potential binding site) and further evaluation keeping the thirty best poses accordingly the London scoring function for binding affinity with a second refinement as a Rigid Receptor using Affinity dG algorithm keeping the ten best poses. The results were analyzed by docking score, frequency of the chemical compound as a stable conformation and the types of interactions at the binding site residues.

### Database preparation

Data repositories such DrugBank, ZINC and PubChem were used to download the dataset from an updated list of reference medicine and The National Compendium of Health Supplies of Mexico (June 2020 update). The structures (sdf) dataset comprises 1,283 unique drugs, which the later were converted into Molecular Database format (mdb) using the Conformation Import MOE, feature available in MOE 2020.09 software (Molecular Operating Environment, Chemical Computing Group, Montreal, Canada) in MOE with an imposed limit of 3 kcal/mol strain energy and a maximum of 250 conformations per molecule and hydrogen atoms were added. Minimum energy configuration was calculated using the Amber10:EHT forcefield.

## Results

### Structural analysis of SARS-CoV-2 – ACE2 interaction

**Figure 1.**
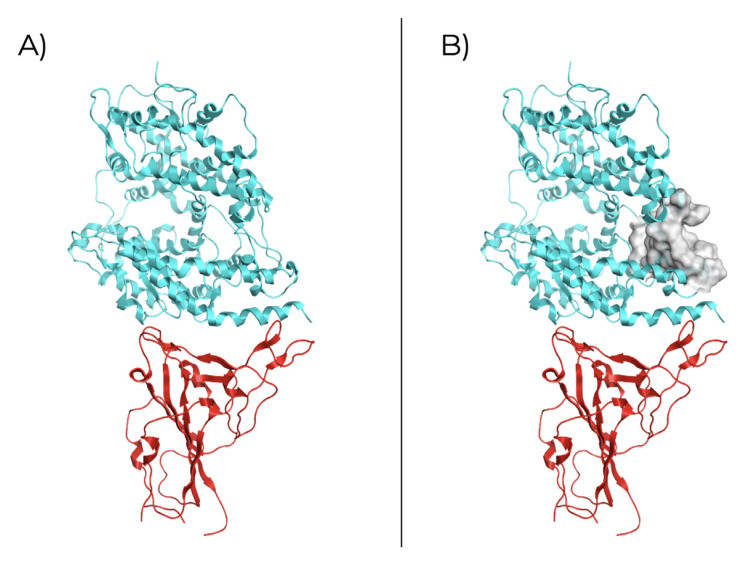
SARS-CoV-2 RBD _S1 _interaction with human ACE2 receptor. **A**) Crystallographic structure (PDB ID: 6VW1) of RBD _S1 _(red) and ACE2 receptor (blue); **B**) Molecular surface of the selected binding site in ACE2.

The structural analysis for the SARS-CoV-2 RBD _S1_ of the spike protein in a complex with the ACE2 (PDB ID: 6VW1; Figure 1A) revealing a potential site for ligand binding inside ACE2 structure ( Table 1). The identified receptor site ( Figure 1B) is proximal to the binding site of RBD _S1_ with a size of 86, therefore it can be used for simulating rigid molecular docking since receptor atoms are in an exposed region of the structure, which could be in favor of drug binding.

**Table 1.** General characteristics of RBD _S1_-ACE2 receptor site.

Size.	PLB	Hyd.	Side	Residues
86	0.84	20	55	Gln81 Tyr83 Pro84 Leu85 Gln86 Leu95 Gln98 Ala99 Gln101 Gln102 Asn103 Ala193 Asn194 His195 Tyr196 Gly205 Asp206 Tyr207 Glu208 Asn210 Arg219 Lys562

### Virtual screening and molecular docking

An average of 78 conformations were generated for each ligand by Conformation Import MOE, generating 100,450 ligand conformations of the FDA approved and prescript drugs by the Mexican Public Health System.

The docking results were sorted and analyzed based on their S score, binding frequency which the drug binds to the receptor site and type of interactions, preferably, hydrogen bond, of the ligand with the selected site.

We selected 38 drugs ( Table 2) that presents the best docking score between −10.04 and −4.04. Subsequently, we shortlisted nine drugs based on their risk of teratogenicity, route of administration, interaction with other drugs, side effects and by their background as pharmacological therapy for the treatment of respiratory diseases.

**Table 2.** List of potential inhibitors of the RBD _S1_-ACE2 interaction.

Drug name	S-Score	Interaction type (number)		
		Hydrogen bond	Ionic bond	Pi-bond
Uridine, trisodium salt	-9.53	10	3	0
Methotrexate sodium	-10.04	8	2	1
Raltritedex	-8.93	8	2	0
Folotyn	-8.19	8	2	0
CDP-choline(1-)	-8.10	8	0	0
Cefuroxime	-8.06	7	2	0
Fexofenadine	-7.89	7	0	0
Fludarabine phosphate	-7.99	6	0	1
Cefixime	-9.02	5	3	0
Aloin	-8.16	5	0	0
Domperidone	-6.63	5	1	0
Tamsulosin	-6.62	5	2	0
Cromoglycic acid	-8.63	4	0	1
Macitentan	-8.06	4	0	2
Tafluprost -Taflutan	-7.94	4	0	1
Thiopental(1-)	-5.42	4	0	0
Metoprolol	-5.10	4	0	0
Irinotecan	-8.72	3	0	1
Pitavastatin(1-)	-8.40	3	0	1
Amlodipine	-5.94	3	0	0
Verapamil	-5.85	3	1	0
Tolterodine	-4.77	3	0	0
Lopinavir	-8.62	2	0	0
Glimepiride	-8.47	2	1	0
Arformoterol	-6.85	2	0	0
Formoterol	-6.15	2	2	1
Ipratropium	-5.38	2	0	1
Pargeverine	-4.53	2	2	0
Pyrilamine	-4.26	2	1	0
Biperiden	-4.23	2	0	1
Orlistat	-8.16	1	0	0
Glyburide	-8.10	1	0	0
Ribociclib	-7.96	1	0	1
Ibesartan	-7.17	1	0	3
Cholecalciferol	-5.81	1	0	0
Testosterone enanthate and estradiol valerate	-5.39	1	0	0
Disopyramide phosphate	-4.22	1	0	2
Primaquine	-4.04	1	1	2

**Figure 2.**
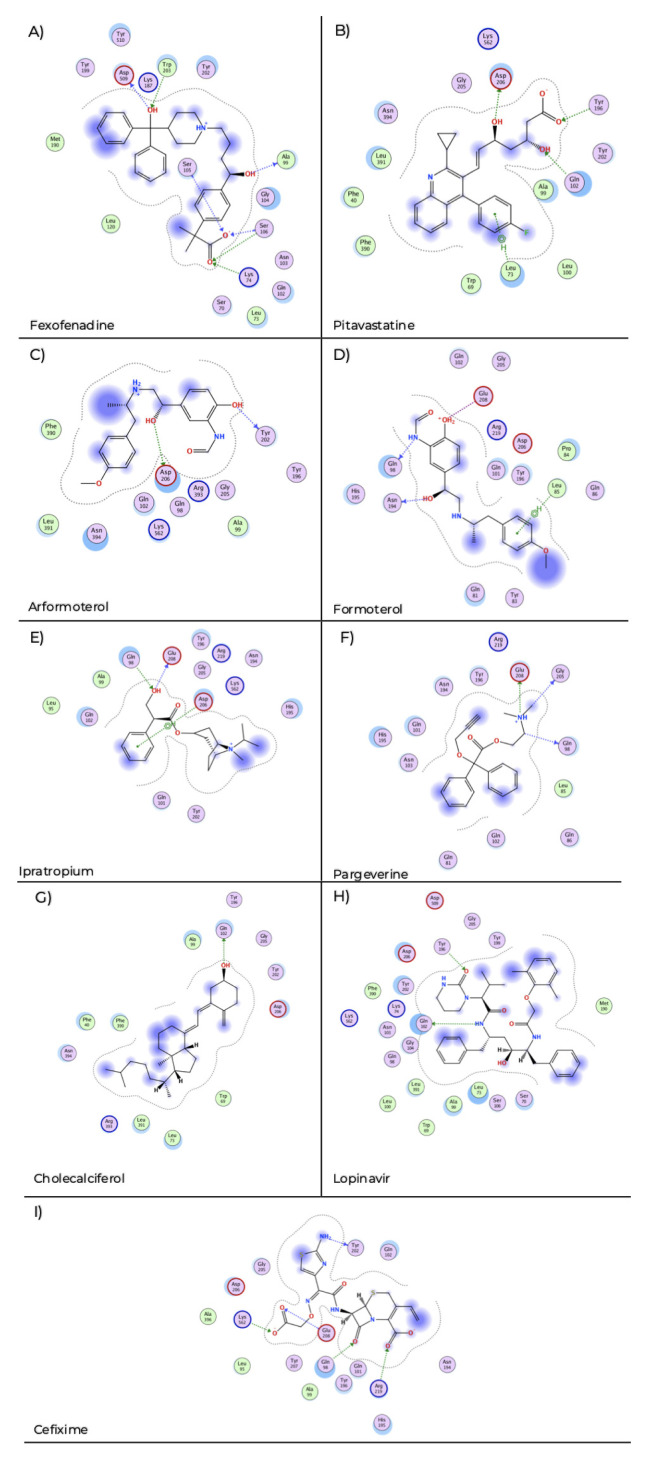
Two-dimensional representation of the interactions of the selected drugs with the ACE2 receptor binding site. The blue arrows indicate the structural hydrogen bridge bonds and the green arrows are the hydrogen bridge bonds with the side chain. **A**) Fexofenadine, **B**) pitavastatine, **C**) aformoterol, **D**) formoterol, **E**) ipatropium, **F**) pargeverine, **G**) cholecalciferol, **H**) lopinavir and **I**) cefixime.

Fexofenadine showed interactions of hydrogen bond with Lys 74, Ala 99, Ser 105, Ser 106, Trp 203 and Asp 509 ( Figure 2A) with a docking score of −7.89. Pitavastatin displays hydrogen bond interaction with Gln 102, Tyr 196, Asp 206 and pi-H stacking with Leu 73 ( Figure 2B) and a docking score of −8.40. Arformoterol showed hydrogen bond interactions with Tyr 202 and Asp 206 ( Figure 2C) with a docking score of −6.85. Formoterol presented hydrogen bond interactions with Gln 98, Asn 194, ionic interaction with Glu 208 and pi-H interaction with Leu 85 ( Figure 2D) and presents a docking score of −6.15. Ipratropium exhibited hydrogen bond interaction with Gln 98, Gln 208 and pi-H stacking with Asp 206 ( Figure 2E) and a docking score of −5.38. Pargeverine shows hydrogen bond interactions with Gln 98, Gly 205, Glu 208 and ionic interaction with Glu 208 ( Figure 2F) and presents a docking score of −4.53. Cholecalciferol presented hydrogen bond interaction with Gln 102 ( Figure 2G) and had a docking score of −5.81. Lopinavir displays hydrogen bond interaction with Gln 102, Tyr 196 ( Figure 2H) and a docking score of −8.62. Cefixime showed hydrogen bond interaction with Gln 98, Tyr 202, Glu 208, Arg 219, Lys 562 and ionic interaction with Arg 219, Lys 562 ( Figure 2I) and a docking score of −9.02.

Some pharmacokinetics characteristics of the shortlisted potential inhibitors of the RBD _S1_ **–**ACE2 interaction are summarized in Table 3 and schematic representations of the interaction maps of these drugs with the selected binding site are shown in Figure 2.

**Table 3.** Potential inhibitors of RBD _S1_-ACE2 interaction selected according to desired characteristics.

Drug	Pharmacokinetics					Route of administration	Drug Type/ Teratogenic risk
	Bioavailability (%)	Protein binding (%)	Metabolism	Half-life (hours)	Excretion		
Fexofenadine	30-41	60-70	Hepatic	14.4	Urine and feces	Oral	Antihistaminic (third generation) /B
Cefixime	30-50	60	Hepatic	3 a 4	Urine and bile	Oral	Antibiotic (Cephalosporin; third generation)/ B
Pitavastatine	60	96	Hepatic	11	Feces	Oral	Statin /X
Lopinavir	25	98-99	Hepatic	2-3	Urine and feces	Oral	Antiviral -Protease inhibitor / C
Arformoterol	21-37	61-64	Hepatic	26	Urine	Inhaled	Long lasting β agonist/ C
Formoterol	61	50	Hepatic	17	Urine	Inhaled	Long lasting β agonist/ C
Ipratropium	2	0-9	Gastrointestinal	1.6	Urine	Inhaled	Anticholinergic bronchodilators / B
Pargeverine	80	90	Hepatic	1.5-2	Urine	Oral, intravenous, intramuscular, rectal	Opium alkaloid antispasmodic / D
Cholecalciferol	NA	NA	Hepatic	NA	Urine	Oral, intramuscular	Vitamin

## Discussion

It has been established that S protein of SARS-CoV-2 virus plays a major role during viral infection. The S protein mediates receptor recognition, cell attachment and fusion of viral membrane with host cell membrane ^24– 29^. The S protein binds to ACE2 receptor through the RBD _S1_, mediating viral attachment to host cell ^30^. ACE2 expression it is distributed mainly in lung, intestine, heart and kidney, also alveolar epithelial type II cells had higher expression levels ^21^. The RBD region is a critical therapeutic target (vaccines and drugs) due to its indispensable function; however, it is suggested that mutations in this region may render pharmacological or immunological therapies ineffective ^31, 32^.

In order to block this event, we propose an *in silico* approach to identify potential inhibitors of the SARS-CoV-2 **–**ACE2 interaction aiming at the ACE2 receptor, blocking the virus accessibility to the membrane-bound ACE2. Here, we screened a drug library consisting of 1300 drugs, FDA approved and prescribed by the Mexican Public Health System, for potential SARS-CoV-2−ACE2 inhibitors, using a rigid receptor docking approach. Utilization of an FDA-approved drug library is an effective and ideal tool for drug repurposing in antiviral research ^33, 34^, such as zika virus ^35^, human rhinovirus ^36^ and hepatitis B virus ^37^. We identify 38 potentially inhibitor drugs of SARS-CoV-2−ACE2 interaction and these are listed on Table 2. Several of those drugs were previously reported to be used for the treatment of respiratory diseases.

Within this list of potential inhibitors of the SARS-CoV-2−ACE2 interaction, is fexofenadine, a third-generation antihistame whose therapeutic indication is the treatment of symptoms of stationary allergies through the selective blockade of H1 receptors ^38^. *In silico* evidence ^39, 40^ suggest that it may interact with the SARS-CoV-2 main protease enzyme M ^Pro^, a key enzyme in viral replication ^41^, acting as a potential inhibitor. Cefixime is a third-generation antibiotic derived from cephalosporin whose use is indicated for the treatment of infections in the upper and lower respiratory tract, otorhinolaryngological ^42^ and urinary tract ^43^, inhibiting the synthesis of the bacterial wall by binding to specific binding proteins for penicillin and is currently used as a secondary therapy to prevent opportunistic infections during the development of COVID-19 ^44^. Pitavastatin is a statin indicated for lowering blood cholesterol levels by inhibiting HMG-CoA reductase, preventing cholesterol synthesis ^45^, and it has also been observed that statin treatments can interfere with viral infectivity through inhibition of glycoprotein processing ^46^ also, they modulates the inflammatory process at cellular level ^47^, which is a remarkable characteristic of the SARS-CoV-2 infection. Additionally, *in silico* findings suggest that could be an efficient inhibitor of SARS-CoV-2 M ^Pro^ ^48^ and SARS-CoV-2 RNA-dependent RNA polymerase (RdRp) ^49^ thru active site binding. Lopinavir is a protease inhibitor indicated as first barrier therapy, in conjunction with Ritonavir, to treat infection caused by the HIV virus by inhibiting the HIV-1 protease ^50^ in addition, studies in cell cultures have shown its effectiveness as an inhibitor of the replication of the MERS-CoV virus ^51^ and SARS-CoV-1 ^52^, while in severe cases of SARS-CoV-2 infection, the results of clinical trials indicate that it is not useful ^53^. Formoterol and aformoterol, anenantiomer of formoterol, are long-lasting selective β agonists indicated for the treatment of chronic obstructive pulmonary disease (COPD) and bronchospasms ^54^, in the same way there is evidence of the use of these drugs as a partial inhibitor of viral replication in primary epithelial cells cultures ^55^ and *in silico* data suggest their binding to the papain-like protease PL _pro_, a coronavirus enzyme essential for viral spread ^56^. Ipratropium is a bronchodilator anticholinergic indicated for the treatment of asthma, shortness of breath, cough and tightness in the chest in patients with COPD ^57, 58^. Inhalation therapy with ipratropium is currently in use to dilate bronchioles in COVID-19 patients to increase oxygen saturation levels (from <80% to 94%) ^59^. Pargeverine is an antispasmodic opioid alkaloid whose therapeutic indication is aimed at the treatment of painful spasms ^60^, also, acts as anticholinergic and has a moderate and non-selective blockade of muscarinic cholinergic fibers ^61^. Since cholinergic activity contribute to airway narrowing, this might be a potential agent to open airway obstruction. Cholecalciferol, is a form of vitamin D (vitamin D3) that can be synthesized naturally in the skin and acts as a hormonal precursor, being converted into calcitriol, and therapeutically is used as a vitamin supplement to treat deficiencies of this vitamin ^62^. In addition, it has been observed that vitamin D supplementation is favorable to reduce viral infections such as influenza ^63, 64^ or more aggressive cases such as HIV ^65^ and it has recently been suggested that it also presents favorable effects before and during the infection caused by SARS-CoV-2 ^66^.

Likewise, it is important to take into account that ACE2 plays an important biological role since regulates cardiovascular functions and innate immune system and, therefore cautions must be taken. Another point to take into account is the delivery method of the drug, since the primary target must be smooth muscle, like the one surrounding the bronchioles, and lung epithelial cells in the airway and airspace compartments, hence, inhalable delivery would be the acceptable choice to deliver the drug in a selectively and localized manner.

Given these characteristics, the results obtained through our *in silico* approach, we consider that the aforementioned drugs are outlined as possible inhibitors of the RBD _S1_-ACE2 interaction. These drugs are well tolerated, commonly used and affordable, hence, most of the drugs on this list can be tested *in vitro*, and even *in vivo* and, consequently, in clinical trials for the development of adjuvant therapies to treat COVID-19.

## Conclusion

In the absence of approved therapies for treatment or prevention, drug repurposing has provided fast and valuable insight into the treatment of COVID-19. Targeting ACE2 receptor as a COVID-19 therapy it is a conceivable approach since it is essential for the viral internalization. However, this approach requires an integrative evaluation of the pros and cons by a clinical context since ACE2 is a multifunctional protein. Jia and collaborators ^23^ present an extensive review for this underexplored approach to treat COVID-19, pointing that it is imperative to determine, by clinicians, the stage of the disease and comorbidities that could prove consequential for an ACE2-targeting regimen. Several drugs are currently investigated by clinical trials or are already in use to treat COVID-19 patients, like lopinavir or ipratropium. In this *in silico* study using structure-bases virtual screening, we identified potential inhibitors of SARS-CoV-2–ACE2 by their interaction with ACE2 receptor. We identify the uridine trisodium salt, methotrexate sodium, raltritedex, folotyn, CDP-choline(1-), cefuroxime, fexofenadine, fludarabine phosphate, cefixime, aloin, domperidone, tamsulosin, cromoglycic acid, macitentan, tafluprost -taflutan, thiopental(1-), metoprolol, irinotecan, pitavastatin(1-), amlodipine, verapamil, tolterodine, lopinavir, glimepiride, arformoterol, formoterol, ipratropium, pargeverine, pyrilamine, biperiden, orlistat, glyburide, ribociclib, ibesartan, cholecalciferol, gravignost, disopyramide phosphate and primaquine. Based on desired characteristics like pharmacokinetics, route of administration or by their background as pharmacological therapy, we propose a shortlist of drugs suitable for testing their potential RBD _S1_ **–**ACE2 inhibitory activity: fexofenadine, cefixime, pitavastatine, lopinavir, arformoterol, formoterol, ipratropium, pargeverine and cholecalciferol. Our identification of potential inhibitors of the SARS-CoV-2–ACE2 interaction among commonly use drugs highlights their potential use for treating COVID-19. Further *in vitro, in vivo* or clinical trial are needed to validate their potential use as inhibitors of SARS-CoV-2–ACE2 interaction.

## Data availability

### Source data

Protein Data Bank: Crystal structure of SARS-CoV-2 receptor binding domain (RBD _S1_) of the spike protein in a complex with the ACE2 receptor. https://identifiers.org/pdb:6vw1

Ministry of Health of Mexico: Drug list used by the Ministry of Health of Mexico (June 2020 version). http://www.csg.gob.mx/Compendio/CNIS/cnis.html.

PubChem: Ligands. https://pubchem.ncbi.nlm.nih.gov.

Drugbank: Ligands. https://go.drugbank.com.

ZINC database: Ligands. http://zinc.docking.org/.

### Extended data

Figshare: Identification of potential inhibitors of SARS-CoV-2 S protein–ACE2 interaction by in silico drug repurposing, https://doi.org/10.6084/m9.figshare.14466333 ^67^

This project contains the underlying data file:

- Table_E1_DrugsAccessionNumber.xlsx (Accession numbers of drugs used for docking simulations)

Data is available under the terms of the Creative Commons Attribution 4.0 International license (CC-BY 4.0)
