# Supplementary material for: Identification of potential inhibitors of SARS-CoV-2 S protein–ACE2 interaction by in silico drug repurposing
Source: F1000Res. 2021 Nov 11;10:Chem Inf Sci-358. Originally published 2021 May 7. [Version 2] doi: 10.12688/f1000research.52168.2 (PMC8630554; doi:10.12688/f1000research.52168.2)
Supplement: Peer review history

# Peer review: Reviewer response for version 2

**DOI:** 10.5256/f1000research.78910.r99909

**Published:** 2021-11-26

**Authors:** Fernando Yepes-Calderon

**Affiliations:** 1 GYM Group SA, Cali, Colombia

No further comments to make.

Is the work clearly and accurately presented and does it cite the current literature?

Partly

If applicable, is the statistical analysis and its interpretation appropriate?

Not applicable

Are all the source data underlying the results available to ensure full reproducibility?

Yes

Is the study design appropriate and is the work technically sound?

Yes

Are the conclusions drawn adequately supported by the results?

Yes

Are sufficient details of methods and analysis provided to allow replication by others?

Partly

Reviewer Expertise:

Medical devices development, Medical Imaging, Methods development

I confirm that I have read this submission and believe that I have an appropriate level of expertise to confirm that it is of an acceptable scientific standard.

# Peer review: Reviewer response for version 2

**DOI:** 10.5256/f1000research.78910.r99910

**Published:** 2021-11-22

**Authors:** Yogendra Nayak

**Affiliations:** 1 Department of Pharmacology, Manipal College of Pharmaceutical Sciences, Manipal Academy of Higher Education, Manipal, India

The revision of the manuscript by the authors looks satisfactory to index the article.

Is the work clearly and accurately presented and does it cite the current literature?

Partly

If applicable, is the statistical analysis and its interpretation appropriate?

Not applicable

Are all the source data underlying the results available to ensure full reproducibility?

Partly

Is the study design appropriate and is the work technically sound?

Partly

Are the conclusions drawn adequately supported by the results?

Partly

Are sufficient details of methods and analysis provided to allow replication by others?

Partly

Reviewer Expertise:

Pharmacy and Pharmacology

I confirm that I have read this submission and believe that I have an appropriate level of expertise to confirm that it is of an acceptable scientific standard.

# Peer review: Reviewer response for version 1

**DOI:** 10.5256/f1000research.55408.r93444

**Published:** 2021-09-21

**Authors:** Yogendra Nayak, Krishnaprasad Baby

**Affiliations:** 1 Department of Pharmacology, Manipal College of Pharmaceutical Sciences, Manipal Academy of Higher Education, Manipal, India; 2 Department of Pharmacology, Manipal College of Pharmaceutical Sciences, Manipal Academy of Higher Education, Manipal, India

I am pleased to write the review for this manuscript. In this manuscript, authors have used computational tools to predict the activity of a suitable drug to treat/ prevent COVID-19. The specific target can prevent the entry of SARS-CoV-2 in human body. Thus keeping the receptor for virus entry ACE2, the available drugs are screened for their binding efficacy, which indirectly correlates the biological activity. Though the computational predictions are widely accepted, this manuscript has few limitations in its current form. Hence, I would suggest minor changes or amendments might be necessary before acceptance. Followings are a few of them:

**Abstract:**

- As the pandemic is dynamically changing the numbers, giving a country specific might not hold good. I suggest to add the data related to the world-wide data can be put.
  An important observation is the number of drugs from US-FDA can be written the same number as it was mentioned later in the manuscript (1,283 may not be correct). 

**Introduction:**

- I feel many places few more additional support from the literature is required. Which can be strengthened by citing references. 

**Methods:**

- Molecule selection for docking appears incorrect. It is protein selection for ligand docking. Moreover, authors have optimised the protein structure, which should have been more appropriate. The resolution of 6VW1 is missing. The rationale for taking  6VW1 appears missing in methodology. 
  Database preparation appears incorrect. It can be dataset/ or ligand preparation. When DrugBank/ZINC15 was accessed is missing (month of accession). Which version of ZINC library was used is missing. Most importantly, the reason why ligands conformation was generated is missing.

**Results:**

- Table 3, the details regarding generation of data appears missing. I feel the detailed legends can be given.
- The current gold standard for molecular activity prediction is MD simulation, this can be tried by authors.

**Discussion:**

- In the methodology the number of drugs selected were 1283, but here it changed to 1300. This looks inconsistent.
- In the methodology, authors have mentioned that they have generated some conformers. Which conformation of drugs were active?
- Overall, I feel the discussion part appears very weak and lacks substantial evidence for how these drugs could be used in COVID-19.

**Conclusions:**

- The conclusion can be made simple by reducing the number of sentences. Most of the journals will not accept citations (references) in conclusions. Hence, the authors can give a thought to modify the conclusion by keeping their views only on objectives/ aims.

Is the work clearly and accurately presented and does it cite the current literature?

Partly

If applicable, is the statistical analysis and its interpretation appropriate?

Not applicable

Are all the source data underlying the results available to ensure full reproducibility?

Partly

Is the study design appropriate and is the work technically sound?

Partly

Are the conclusions drawn adequately supported by the results?

Partly

Are sufficient details of methods and analysis provided to allow replication by others?

Partly

Reviewer Expertise:

Pharmacy and Pharmacology

We confirm that we have read this submission and believe that we have an appropriate level of expertise to confirm that it is of an acceptable scientific standard, however we have significant reservations, as outlined above.

# Response

**Published:** 2021-10-29

**Authors:** Guillermo A Silva-Martinez

**Affiliations:** Tecnologico Nacional de Mexico en Celaya, Mexico

**We thank the reviewer for the detailed revision of our paper and for suggesting improvements.**

**Abstract:**

- *As the pandemic is dynamically changing the numbers, giving a country specific might not hold good. I suggest to add the data related to the world-wide data can be put.*
- *An important observation is the number of drugs from US-FDA can be written the same number as it was mentioned later in the manuscript (1,283 may not be correct). *

**Response: We thank the reviewer for this suggestion to improve the impact of our abstract. US-FDA approved drug number was corrected.**

**Introduction:**

- *I feel many places few more additional support from the literature is required. Which can be strengthened by citing references. *

**Response: We agree with the reviewer's observation, however, we believe that the introduction provides the necessary information to be in context to the overall objective of our work.**

**Methods:**

- *Molecule selection for docking appears incorrect. It is protein selection for ligand docking. Moreover, authors have optimized the protein structure, which should have been more appropriate. The resolution of 6VW1 is missing. The rationale for taking 6VW1 appears missing in methodology. *
* Database preparation appears incorrect. It can be dataset/ or ligand preparation. When DrugBank/ZINC15 was accessed is missing (month of accession). Which version of ZINC library was used is missing. Most importantly, the reason why ligands conformation was generated is missing.*
**Response: We thank the reviewer for the comments and for suggesting improvements. Changes on the subsections mentioned above were made. Resolution of 6VW1, the rationale of using that structure and accession date to databases were added to the manuscript. In the “Protein selection for ligand docking”, it is now clearly specified that rigid molecular docking is carrying out. In this type of docking, protein orientation is fixed and only ligands are allowed to vary their orientation, therefore each conformer allow to simulate this variation. We assumed that no further explanation was needed. **

**Results:**

- *Table 3, the details regarding generation of data appears missing. I feel the detailed legends can be given.*
**Response: We shortlisted nine drugs based on their risk of teratogenicity, route of administration, interaction with other drugs, side effects and by their background as pharmacological therapy for the treatment of respiratory diseases. It is described in the Results section, subsection Virtual Screening and Molecular docking, paragraph 3.**
- *The current gold standard for molecular activity prediction is MD simulation, this can be tried by authors.*
**Response: We agree with the reviewer comment. Unfortunately, we do not have the computational infrastructure or access to supercomputer cluster to run MD. We are trying to run MD on our workstations, but those simulations will take time. However, we feel that our work is a valid approximation for COVID19 drug repurposing.**

**Discussion:**

- *In the methodology the number of drugs selected where 1283, but here it changed to 1300. this looks in consistency.*
** Response: We thank reviewer comment. See response above.**
- *In the methodology, authors have mentioned that they have generated some conformers. Which conformation of drugs were active?*
**Response: In order to generate active conformers for docking simulation, we impose a 3 kcal/mol limit on strain energy, which means that conformation elucidates under these criteria can occur under physiological conditions and maintain their activity. Methodology section was restructured to be more clear.**
- *Overall, I feel the discussion part appears very weak and lacks substantial evidence for how these drugs could be used in COVID-19.*
**Response: We thank the reviewer for this comment. We feel that most of the relevant evidence -to date of manuscript submission- regarding COVID19 and the potential inhibitors, were discussed.**

**Conclusions:**

- *The conclusion can be made simple by reducing the number of sentences. Most of the journals will not accept citations (references) in conclusions. Hence, the authors can give a thought to modify the conclusion by keeping their views only on objectives/ aims.*
**Response: We thank the reviewer for this observation. This section was restructured.**

# Peer review: Reviewer response for version 1

**DOI:** 10.5256/f1000research.55408.r85900

**Published:** 2021-06-18

**Authors:** Fernando Yepes-Calderon

**Affiliations:** 1 GYM Group SA, Cali, Colombia

Authors use molecular docking tools to test existing medicine in their efficacy to impede the S protein-ACE2 binding and thus, disabling the entry point of Cov2 to human cells.

 The authors use a solid justification to support this project. Since medicine creation is an expensive and long-lasting task, verifying if some active molecules in already FDA-approved medicine might block the binding between the S-protein and the ACE2 is an excellent alternate path.

 The document is generally well organized and pleasant to read. I suggest adding some corrections to the abstract as follows.

**Background** 

 A new coronavirus outbreak, firstly reported in Wuhan, China, in December 2019, causes COVID-19, which symptoms are similar to SARS-atypical pneumonia. Worldwide, around 116 million cases and 2.57 million deaths are reported, with new cases increasing mortality every day. To date, there is no specific commercial treatment to control the infection.

 Repurpose drugs targeting the angiotensin-converting enzyme 2 (ACE2) receptor represent an alternative strategy to block the binding of CoV2's protein S and forestall virus adhesion, internalization, and replication in the host cell.

**Methods**

 We performed rigid molecular docking using the receptor-binding domain at the S1 subunit of the S protein (RBDS1)-ACE2 (PDB ID:6VW1) interaction site and 1,283 FDA-approved drugs. The docking score, frequency of the drug in the receptor site, and interactions at the binding site residues were used as analyzing criteria.

**Results**

 This research yielded 40 drugs labeled as potential inhibitors of RBD S1-ACE2 interaction. Among the inhibitors, compounds such as ipratropium, formoterol, and fexofenadine can be found. Specialists employ these drugs to treat chronic obstructive pulmonary diseases, asthma, and almost any respiratory infection.

 Conclusions: Our results will serve as the basis for *in vitro* and *in vivo* studies to evaluate the potential use of those drugs to generate

 affordable and convenient therapies to treat COVID-19.

 The proposed modifications focus on keeping the communications global. The CoV2 numbers in Mexico are absent in this suggested abstract since the authors provide worldwide numbers. I also shortened some sentences and fixed some wrongly used singulars.

1. Correct the hyphenation in the whole document. If the authors are using a latex template, use the command \hyphenation{co-rrect, se-pa-ra-tion, sche-me}.
  Events: Janu-ary and associ-ated  (intro, paragraph 2), lig-and (methods, paragraph 2).
2. Introduction, page 3, paragraph 2.  Current events suggest that Covid19 is not only lethal in the elder or patients with chronic diseases. I might suggest deleting this statement.
3. Introduction, page 3, paragraph 2. something is missing in this paragraph. Also, the statement may be misleading; the actual spread and high contamination levels are proof of non-efficiency. If isolation and the other measurements were effective, what would be the sense for the proposal?
4. In subsection "Database preparation," you have information already provided in the first subsection of the methods.
5. Discussion, page 7, paragraph 1. In "ACE2 expression it is" the authors might have two nouns in the same sentence.

Regarding the methodology, the authors used a standard: importing the macromolecule, edit or delete water molecules, add hydrogens, add charges, and the ligand.  Did the authors have any particular issue during the process? In case affirmative, add it to assert reproducibility.

 The list of potential inhibitors is highly appreciated. The authors presented the interactions of the selected drugs nicely.

 I suggest the authors reinforcing their discussion with arguments derived from the mutation capacity of the retrovirus that might render current vaccination not entirely compelling. Therefore, the presented alternatives could set as plausible options for treatment.

Is the work clearly and accurately presented and does it cite the current literature?

Partly

If applicable, is the statistical analysis and its interpretation appropriate?

Not applicable

Are all the source data underlying the results available to ensure full reproducibility?

Yes

Is the study design appropriate and is the work technically sound?

Yes

Are the conclusions drawn adequately supported by the results?

Yes

Are sufficient details of methods and analysis provided to allow replication by others?

Partly

Reviewer Expertise:

Medical devices development, Medical Imaging, Methods development

I confirm that I have read this submission and believe that I have an appropriate level of expertise to confirm that it is of an acceptable scientific standard, however I have significant reservations, as outlined above.

# Response

**Published:** 2021-10-29

**Authors:** Guillermo A Silva-Martinez

**Affiliations:** Tecnologico Nacional de Mexico en Celaya, Mexico

**We thank you for the detailed revision of our paper and for suggesting improvements. Also, we want to apologize for the long delay on our response, we were waiting for a second reviewer report to edit our manuscript.**

*Authors use molecular docking tools to test existing medicine in their efficacy to impede the S protein-ACE2 binding and thus, disabling the entry point of Cov2 to human cells.*

*The authors use a solid justification to support this project. Since medicine creation is an expensive and long-lasting task, verifying if some active molecules in already FDA-approved medicine might block the binding between the S-protein and the ACE2 is an excellent alternate path.*

*The document is generally well organized and pleasant to read. I suggest adding some corrections to the abstract as follows.*

**Response: We thank the reviewer for the detailed revision of our paper and for suggesting improvements. We are pleased that you found the structure of the writing to be pleasant.**

***Background***

*A new coronavirus outbreak, firstly reported in Wuhan, China, in December 2019, causes COVID-19, which symptoms are similar to SARS-atypical pneumonia. Worldwide, around 116 million cases and 2.57 million deaths are reported, with new cases increasing mortality every day. To date, there is no specific commercial treatment to control the infection.*

*Repurpose drugs targeting the angiotensin-converting enzyme 2 (ACE2) receptor represent an alternative strategy to block the binding of CoV2's protein S and forestall virus adhesion, internalization, and replication in the host cell.*

***Methods***

*We performed rigid molecular docking using the receptor-binding domain at the S1 subunit of the S protein (RBDS1)-ACE2 (PDB ID:6VW1) interaction site and 1,283 FDA-approved drugs. The docking score, frequency of the drug in the receptor site, and interactions at the binding site residues were used as analyzing criteria.*

***Results***

*This research yielded 40 drugs labeled as potential inhibitors of RBD S1-ACE2 interaction. Among the inhibitors, compounds such as ipratropium, formoterol, and fexofenadine can be found. Specialists employ these drugs to treat chronic obstructive pulmonary diseases, asthma, and almost any respiratory infection.*

*Conclusions: Our results will serve as the basis for in vitro and in vivo studies to evaluate the potential use of those drugs to generate affordable and convenient therapies to treat COVID-19.*

- **Response: Taking into account your contribution, in the manuscript the abstract has been improved and better organized.**

The proposed modifications focus on keeping the communications global. The CoV2 numbers in Mexico are absent in this suggested abstract since the authors provide worldwide numbers. I also shortened some sentences and fixed some wrongly used singulars. 

  

1. *Correct the hyphenation in the whole document. If the authors are using a latex template, use the command \hyphenation{co-rrect, se-pa-ra-tion, sche-me}.*
* Events: Janu-ary and associ-ated  (intro, paragraph 2), lig-and (methods, paragraph 2).*
**Response: We thank the reviewer for this observation. The hyphenation has been corrected in the whole manuscript.**
2. *Introduction, page 3, paragraph 2.  Current events suggest that Covid19 is not only lethal in the elder or patients with chronic diseases. I might suggest deleting this statement*.
**Response: We agree with the reviewer's observation, however it’s important to remark that adult individuals (over 40 years) and patients with comorbidities are prone to death by COVID 19 disease. This paragraph was restructured.**
3. *Introduction, page 3, paragraph 2. something is missing in this paragraph. Also, the statement may be misleading; the actual spread and high contamination levels are proof of non-efficiency. If isolation and the other measurements were effective, what would be the sense for the proposal?*
**Response: We thank the reviewer for this comment. This paragraph was rewritten.**
4. *In subsection "Database preparation," you have information already provided in the first subsection of the methods.*
**Response: We thank to reviewer for this comment. Changes were made in this section.**
5. *Discussion, page 7, paragraph 1. In "ACE2 expression it is" the authors might have two nouns in the same sentence.*
**Response: We thank the reviewer for this comment. Changes were made in this section.**

*Regarding the methodology, the authors used a standard: importing the macromolecule, edit or delete water molecules, add hydrogens, add charges, and the ligand.  Did the authors have any particular issue during the process? In case affirmative, add it to assert reproducibility.*

**Response: We do not have any issue during the structure preparation; therefore, reproducibility can be achieved without any problem.**

*The list of potential inhibitors is highly appreciated. The authors presented the interactions of the selected drugs nicely.*

**Response: We appreciate that the reviewer finds our data well-presented.**

*I suggest the authors reinforcing their discussion with arguments derived from the mutation capacity of the retrovirus that might render current vaccination not entirely compelling. Therefore, the presented alternatives could set as plausible options for treatment.*

**Response: We thank the reviewer for suggesting improvements to our discussion section. Reinforced of the discussion section were made in the manuscript taking reviewers suggestion.**
